# High-Precision Detection of Earth’s Free Oscillation Signals with Consideration of Phase Consistency

**DOI:** 10.3390/s26020492

**Published:** 2026-01-12

**Authors:** Yaxin Zhao, Gong Xu, Hanwei Zhang, Xiuhua Sun

**Affiliations:** 1 School of Civil Engineering and Spatial Information, Shandong University of Technology, Zibo 255000, China; 2 Zibo Land Survey and Mapping Co., Ltd., Zibo 255000, China

**Keywords:** NTFT, OSE, phase consistency, asynchronous oscillations, normal models detection

## Abstract

High-precision detection of normal modes is crucial for revealing Earth’s deep internal structure. Using superconducting gravimeter data, this study achieves high-precision normal mode detection by combining Normal Time-Frequency Transform (NTFT) and Optimal Sequence Estimation (OSE). Experiments show that OSE detection results vary significantly with the number of stations or different station combinations, indicating the existence of normal mode asynchronous oscillations that notably impact OSE accuracy. NTFT is then applied to extract each station’s instantaneous phase spectrum, confirming asynchronous oscillations and highlighting the necessity of considering phase consistency in OSE-based detection. Finally, by integrating NTFT and OSE, a high-precision detection method that accounts for phase consistency is proposed. For the _3_S_1_ model, the singlet frequencies of the *m* = −1, 0, and 1 states were detected to be 0.9424 mHz, 0.944 mHz, and 0.9455 mHz, respectively. The detection results are in excellent agreement with the PREM theoretical values, which validates the effectiveness of the proposed method. This research provides foundational data and key technical support for further exploring Earth’s deep structure and optimizing Earth models.

## 1. Introduction

Earth’s free oscillations (normal modes) are global vibrational phenomena that occur after the Earth experiences a significant disturbance, such as a strong earthquake. The oscillation frequency and amplitude contain key information about the Earth’s internal structure and source mechanism. Precise frequency detection is crucial for inverting the Earth’s deep-layered structure. Therefore, studying the modal and splitting characteristics of normal modes is a core task for analyzing relevant geophysical processes and revealing the internal structure of the Earth [1]. Normal modes play a significant role in constructing detailed models of the Earth’s interior and in enhancing the understanding of deep Earth dynamic processes, with important scientific value [2,3,4,5,6,7].

Precise detection of normal modes frequencies is closely related to the Earth’s internal density and can provide important constraints on the Earth’s layered structure [8,9,10,11,12,13]. On one hand, in seismological research, the frequency of normal modes is one of the essential parameters for inverting seismic moment tensors and source mechanisms, and for determining the rupture process of an earthquake [8]. On the other hand, the frequency of normal modes is also an important method for testing the accuracy of existing Earth models, such as the PREM model [9]. Furthermore, the multi-mode frequencies of normal modes reflect the lateral heterogeneity of material composition, temperature, or pressure in the deep Earth, such as anomalies in the mantle transition zone or anisotropy in the inner core. Taking the _3_S_1_ model studied in this paper as an example, its splitting information can constrain the structure and density of the Earth’s inner core [8,10]. Therefore, high-precision detection of normal modes frequencies is crucial for the study of the Earth’s deep internal structure [14,15,16].

It is difficult for a single station to detect the weak amplitude of normal modes signals. By stacking seismic data from multiple sources, the signal-to-noise ratio can be improved, allowing for the extraction of the target signal [17,18,19,20,21,22,23,24,25]. Currently, detection of normal modes based on stacking methods has become relatively mature. The underlying assumption of these methods is global synchronous oscillation [19,20,21,22,23,24,25], meaning that after the Earth is disturbed, the normal modes signals at any location on Earth are simultaneously excited and continuously oscillate. The use of multiple stations for detection amplifies the signal. Chao et al. [23] detected the frequencies of the _3_S_1_ model with *m* = −1, 0, 1 modes as 0.9427 mHz, 0.94535 mHz, and 0.94563 mHz, respectively, based on the SHS method. Roult et al. [24] obtained the model frequencies as 0.94256 mHz, 0.94419 mHz, and 0.94579 mHz using the MSE method. Ding Hao [19] detected the average frequencies of the *m* = −1 and 1 modes of the model as 0.94254 mHz and 0.94575 mHz, respectively, using the PSA method.

Based on the global synchronous assumption, it is generally believed that the more stations used in stacking methods, the higher the detection accuracy [19,20,21,22,23,24,25]. However, there are discrepancies in the frequency detection results for the _3_S_1_ model among different researchers. Ding Hao [19] detected the frequencies of the *m* = −1, 0, 1 modes for this model as 0.942267 mHz, 0.944217 mHz, and 0.945472 mHz using the OSE method, while when different station combinations were used, the detected frequencies were 0.942557 mHz, 0.944765 mHz, and 0.945763 mHz [20]. Majstorovic [22], using the OSE method, detected the model frequencies as 0.942565 mHz, 0.94457 mHz, and 0.945472 mHz. Apart from stacking methods, Shen et al. [25] used the EEMD method and detected the frequencies of the _3_S_1_ model’s *m* = −1, 0, 1 modes as 0.942598 mHz, 0.944113 mHz, and 0.945864 mHz, respectively. Zhang Yingqi et al. [26] used the FFT method and detected the average frequencies for the m = −1 and 1 modes of this model as 0.94271 mHz and 0.94575 mHz. Chao et al. [23], using the AR method, detected the frequencies as 0.9427 mHz, 0.94535 mHz, and 0.94563 mHz, respectively. These discrepancies may suggest that the assumption of global synchronous oscillations is controversial.

This controversy may indicate the existence of asynchronous oscillations in the Earth, a phenomenon presumably attributed to the influence of lateral inhomogeneities in the Earth’s interior. The Earth is not an ideal homogeneous sphere; its deep layers exhibit significant lateral variations in material composition, temperature, and pressure [8,10]. When free oscillation waves propagate through an inhomogeneous medium, the wave velocities along different propagation paths undergo variations due to differences in medium properties, which in turn gives rise to phase differences in the oscillation signals received by various seismic stations. Furthermore, the viscoelastic properties of the Earth’s internal medium induce attenuation of free oscillation signals, with attenuation coefficients varying across different regions. Such attenuation discrepancies accumulate over time, further exacerbating the phase inconsistency among stations [17,18,19,20,21,22,23,24,25,26].

To further investigate this phenomenon, this paper introduces time-frequency analysis to determine the phase information of normal modes, thereby revealing the phase consistency of normal modes. Based on this, a high-precision normal models detection method that takes phase consistency into account is proposed, combining NTFT and OSE. This method not only considers phase consistency but also leverages the advantage of OSE in detecting weak signals, providing higher accuracy in normal modes detection. This offers an innovative approach to high-precision normal modes detection and provides more precise constraints for the study of Earth’s deep internal structure.

## 2. Normal Modes Frequency and Phase Detection Methods

### 2.1. Stacking Methods

Common stacking methods include linear stacking, weighted stacking, spherical harmonic stacking (SHS), multi-station experiment techniques (MSE), and optimal sequence estimation (OSE), among others. Many scholars have made comparative analyses of these methods. Among them, Ding et al. [18] have verified that the detection results using the OSE method are superior to those obtained using SHS and MSE. Therefore, this paper uses the OSE method to detect the frequencies of normal modes.

Considering only the radial component of the surface displacement in a viscoelastic Earth model, the station on the Earth’s surface, denoted as Ω=(θ,ϕ) and (θ,ϕ) for the colatitude and longitude, respectively, has a radial displacement $u_{R}$ given by [22]:

(1) uR(Ω,t)=∑mUlnYlm(Ω)sm(r0)eiσmt

In the equation, *t* represents time, Uln is the radial eigenfunction at the Earth’s surface, $Y_{l}^{m}(\Omega )$ is the spherical harmonic function, $s_{m}(r_{0})$ represents the source vector of each mode excitation, which is determined by the seismic moment tensor and the strain tensor, $r_{0}$ denotes the location of the source, and $\sigma_{m}$ represents the complex eigenfrequency of the mode.

Introducing the symbols ϵm=Ulnsm(r0) and $A_{m}=\epsilon_{m}e^{i\sigma_{m}t}$, Equation (1) simplifies to:

(2) uR(Ω,t)=∑mUlnYlm(Ω)sm(r0)eiσmt

(3) $${\left[\begin{array}{ccccc} u_{R}(\Omega_{1},t) & =A_{-l}Y_{l}^{-l}(\Omega ) & +A_{-l+1}Y_{l}^{-l+1}(\Omega ) & +\;\ldots \;+ & A_{l}Y_{l}^{l}(\Omega )\\ u_{R}(\Omega_{2},t) & =A_{-l}Y_{l}^{-l}(\Omega ) & +A_{-l+1}Y_{l}^{-l+1}(\Omega ) & +\ldots + & A_{l}Y_{l}^{l}(\Omega )\\ \vdots & & & \vdots & \\ u_{R}(\Omega_{N},t) & =A_{-l}Y_{l}^{-l}(\Omega ) & +A_{-l+1}Y_{l}^{-l+1}(\Omega ) & +\ldots + & A_{l}Y_{l}^{l}(\Omega ) \end{array}\right]}_{N\times (2l+1)}$$

Let $\Omega_{j}(j=1,2,\ldots ,N)$ represent the position of the *j*-th superconducting gravimeter station. Then, the stacking of *N* stations can be expressed as follows:

(4) U=YA

(5) $$U={\left[\begin{array}{c} u_{R}(\Omega_{1},t)\\ u_{R}(\Omega_{2},t)\\ \vdots \\ u_{R}(\Omega_{N},t) \end{array}\right]}_{N\times x}$$

(6) $${\left.Y=\left[\begin{array}{cccc} Y_{L}^{-L}(\Omega_{1}) & Y_{L}^{-L+1}(\Omega_{1}) & \ldots & Y_{L}^{L}(\Omega_{1})\\ Y_{L}^{-L}(\Omega_{2}) & Y_{L}^{-L+1}(\Omega_{2}) & \ldots & Y_{L}^{L}(\Omega_{2})\\ \vdots & & \vdots & \\ Y_{L}^{-L}(\Omega_{N}) & Y_{L}^{-L+1}(\Omega_{N}) & \ldots & Y_{L}^{L}(\Omega_{N}) \end{array}\right.\right]}_{N\times (2l+1)}$$

(7) $$A={\left[\begin{array}{c} \epsilon_{-L}\exp^{i\sigma_{-L}t}\\ \epsilon_{-L+1}\exp^{i\sigma_{-L+1}t}\\ \vdots \\ \epsilon_{L}\exp^{i\sigma_{L}t} \end{array}\right]}_{\left(2l+1\right)\times x}$$

In the equation, *t* represents the time samples from 1 to *x*, U is the *N* × *x* radial component observation matrix of the superconducting gravimeters, *Y* is the *N* × (2*l* + 1) spherical harmonic function matrix, and *A* is the matrix to be inverted, with each row representing a single-mode of normal modes. When *N* > 2*l* + 1, the least squares method can be used to obtain:

(8) $$A={\left(Y^{T}PY\right)}^{-1}Y^{T}PU$$

To account for the observation quality of each station, *P_j_* in the equation represents the weight matrix, which is inversely proportional to the station’s signal-to-noise ratio.

### 2.2. Unbiased Phase Detection Based on NTFT

NTFT can unbiasedly determine the instantaneous amplitude, instantaneous period, and instantaneous phase of periodic signals, offering certain advantages in extracting non-stationary and nonlinear signals. Based on standard time-frequency transform theory, this paper provides a detailed argument that NTFT can unbiasedly determine the phase of normal modes, thereby laying the theoretical foundation for applying this method to detect the phase information of normal modes.

(1) NTFT Theory

The standard time-frequency transform of the function $f(t)\in L^{1}(R)$ is defined as [26,27,28,29]:

(9) $$\Psi f(\tau ,\varpi )=\int_{R}f(t)\bar{\psi (t-\tau ,\varpi )}dt,\tau ,\varpi \in R$$

In the equation, τ is the time factor, ϖ is the frequency factor, the overline “-“ represents the conjugate operator, and Ψ(t,ϖ) is the kernel function of the time-frequency transform, satisfying:

(10) $$\hat{\psi }(\omega ,\varpi )=\int_{R}\psi (t,\varpi )\exp(-i\omega t)dt\dot{\neq }0$$

In the equation, “·” denotes “almost everywhere”, and ω represents the frequency factor.

A typical NTFT kernel function is:

(11) $$\psi (t,\varpi )=|\mu (\varpi )|w(\mu (\varpi )t)exp(i\varpi t),w(t)\in \Omega (R),(\mu (\varpi )\in R)\dot{\neq }0$$

In the equation, w(t) represents the window function.

The NTFT of the time function $h\left(t\right)=A\exp\left(i\left(\varphi +\beta t\right)\right)$ satisfies:

(12) $$\left|\begin{array}{c} \Psi h(\tau ,\varpi ) \end{array}\right|=\mathrm{Maximun}\Leftrightarrow \varpi =\beta ,\forall \tau \in R$$

(13) Ψh(τ,β)=h(τ)=|A|exp(i(ϕ+βτ)),∀τ∈R

Equation (12) shows that NTFT can unbiasedly determine the instantaneous frequency of the signal, and Equation (13) can unbiasedly determine the instantaneous amplitude and instantaneous phase of the signal.

(2) Unbiased Phase Detection

The normal modes theoretical model can be expressed as the sum of damped oscillatory cosine signals, with each signal represented as follows:

(14) $$u(t)=\sum_{k}\left|A_{k}|\exp(-\alpha_{k}t)\cos(\beta_{k}t+\phi_{k}\right)$$

$\left|A_{k}\right|$ is the amplitude, $\alpha_{k}$ is the damping factor, $\beta_{k}$ is the angular frequency, and $\phi_{k}$ is the initial phase. According to Euler’s theorem, we can obtain:

(15) $$u(t)=\frac{1}{2}(O(t)+\bar{O(t)})$$

In the equation, O(t)=|A|exp(−αt)exp(i(βt+ϕ)) is the complex signal of u(t), and the overline represents the conjugate operator.

The NTFT kernel function ψ(t,ϖ)=w(t)exp(iϖt), where $w(t)=\frac{1}{\sqrt{2\pi }\sigma }\exp(-\frac{t^{2}}{2\sigma^{2}}),\sigma >0$ is the standard Gaussian window function, then the NTFT of O(t) and $\bar{O(t)}$ is:

(16) $$\Psi O(\tau ,\varpi )=O(\tau )\exp\left(\frac{\sigma^{2}\alpha^{2}}{2}-\frac{\sigma^{2}{\left(\varpi -\beta \right)}^{2}}{2}\right)\exp\left(i\sigma^{2}\alpha (\varpi -\beta )\right)$$

(17) $$\Psi \bar{O(\tau ,\varpi )}=\bar{O(\tau )}\exp\left(\frac{\sigma^{2}\alpha^{2}}{2}-\frac{\sigma^{2}{\left(\varpi +\beta \right)}^{2}}{2}\right)\exp\left(i\sigma^{2}\alpha (\varpi +\beta )\right)$$

From the properties of the standard Gaussian window function, it is known that the NTFT of ϖ=β,(ϖ>0), $\bar{O(t)}$ is almost zero. Therefore, the NTFT of the normal modes displacement u(t) can be expressed as follows:

(18) $$\Psi u(\tau ,\varpi )=\frac{1}{2}\Psi O(\tau )=\frac{1}{2}O(\tau )\exp\left(\frac{\sigma^{2}\alpha^{2}}{2}-\frac{\sigma^{2}{\left(\varpi -\beta \right)}^{2}}{2}\right)\exp\left(i\sigma^{2}\alpha (\varpi -\beta )\right)$$

The corresponding NTFT spectrum is:

(19) $$|\Psi u(\tau ,\varpi )|=\frac{1}{2}|A|\exp(-\alpha \tau )\exp\left(\frac{\sigma^{2}\alpha^{2}}{2}-\frac{\sigma^{2}{\left(\varpi -\beta \right)}^{2}}{2}\right)$$

From Equation (19), it can be seen that when ϖ=β, the NTFT spectrum reaches its maximum value. This theoretically ensures that the NTFT spectrum can unbiasedly reveal the frequency of the single mode of normal modes.

Substituting β into Equation (18) gives:

(20) $$\Psi u(\tau ,\beta )=\frac{1}{2}O(\tau )\exp\left(\frac{\sigma^{2}\alpha^{2}}{2}\right)$$

Equation (20) shows that the NTFT coefficient is essentially the normal modes multiplied by a real number $\frac{1}{2}\exp\left(\frac{\sigma^{2}\alpha^{2}}{2}\right)$. Since a real number does not alter the phase of the normal modes, the NTFT coefficient can unbiasedly reveal the instantaneous phase of the signal.

## 3. Asynchronous Oscillation Verification

### 3.1. Data Preparation

**Figure 1:**
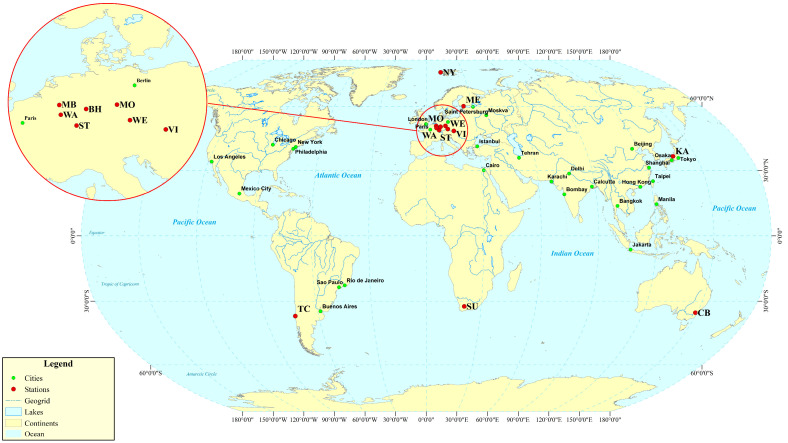
Station Distribution Map.

This study selects the weak signal of the _3_S_1_ mode excited by the 2004 Sumatra Earthquake (Mw 9.0) as the research object. The superconducting gravimeter observation data from all 17 stations were obtained from the International Geodynamics and Earth Tide Service (IGETS) [30], with a sampling interval of 1 min. Since the _1_S_3_ model affects the detection of the _3_S_1_ model within 50 h after the earthquake [31], the analysis was conducted on data from 50 to 280 h post-earthquake, with 280 h being the optimal length suggested by Dahlen [32] corresponding to 1.1 times the quality factor. The station distribution is shown in Figure 1, and station information is provided in Table 1.

In this paper, Level 3 data obtained from IGETS (ftp://igetsftp.gfz-potsdam.de, accessed on 2 November 2024) [30] is used. This data has been corrected for solid tide, ocean tide loading, atmospheric loading, Earth’s rotation, instrument drift, and other factors, achieving microgal-level precision.

### 3.2. OSE Method’s Dependence on the Number and Combination of Stations

**Figure 2:**
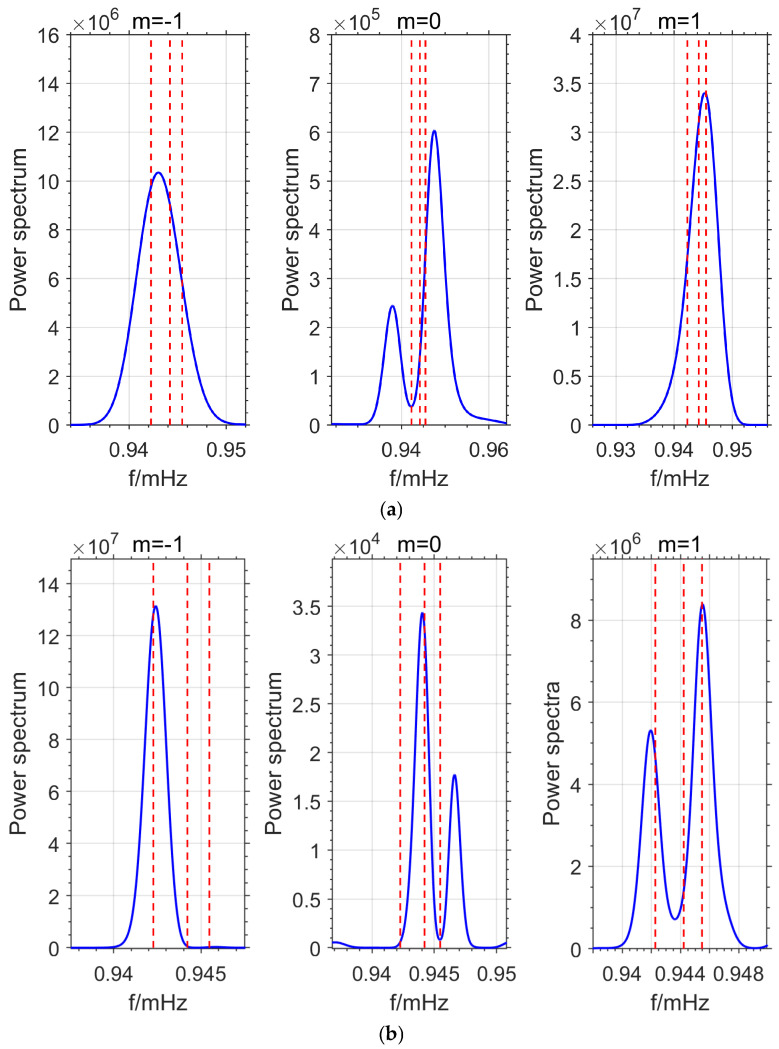
OSE Detection Results. (**a**) Detection Results for 17 Stations. (**b**) Detection Results for 5 Stations.

From Equation (8), it can be concluded that, under the assumption of consistent oscillation, the more excess observations there are, the higher the detection accuracy. To explore the relationship between the OSE method and the number and combination of stations used, the following experiment was designed. Based on Equation (8), OSE stacking detection experiments were conducted using all 17 stations and 5 stations (ST, MO-2, WA, SU-2, and WE-2), respectively. The results are shown in Figure 2. The three red dashed lines in the figure (from left to right) correspond to the three single-mode PREM theoretical frequencies of the _3_S_1_ model for *m* = −1, 0, and 1, which are 0.94227 mHz, 0.94422 mHz, and 0.94547 mHz, respectively.

As shown in Figure 2a, the 17 stations did not detect the single-mode signal for *m* = 0. The frequency of the detected single-mode for *m* = −1 was 0.943 mHz, and for *m* = 1, it was 0.9453 mHz. The deviations from the PREM theoretical values were 0.733 μHz and −0.172 μHz, respectively. In Figure 2b, the detected single-mode frequencies for *m* = −1, 0, and 1 were 0.9424 mHz, 0.944 mHz, and 0.9455 mHz, with deviations from the PREM theoretical values of 0.133 μHz, 0.285 μHz, and 0.028 μHz, respectively. The detection results are in good agreement with the theoretical values, particularly for the *m* = 1 single-mode, where the detected frequency is almost identical to the theoretical value of 0.94547 mHz. In theory, stacking data from more stations should effectively reduce random noise and enhance the signal, making the detection results more accurate as the number of stations increases. However, as seen from the experimental results in Figure 2, the actual detection outcome does not necessarily improve with an increasing number of stations.

**Figure 3:**
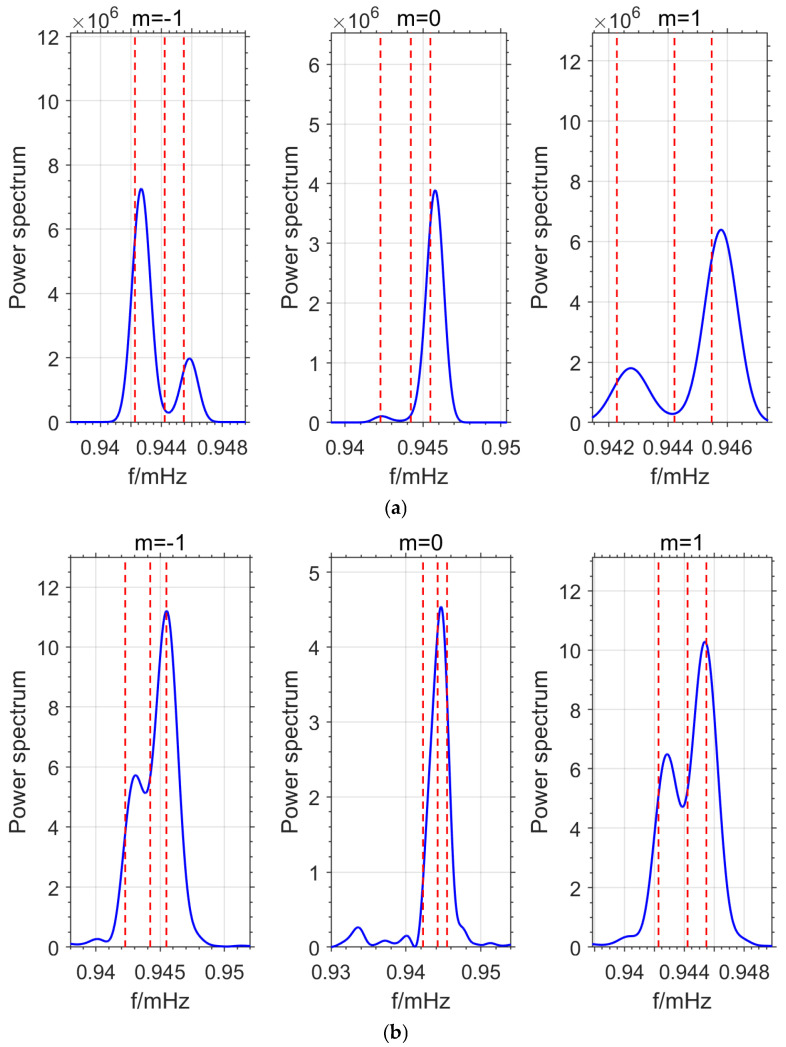
Experimental Results Exploring the Relationship Between Stacking Methods and Station Distribution. (**a**) Stacking Results for ST, MO-2, WA, BH-2, CB Stations. (**b**) Stacking Results for MO-1, SU-1, BH-1, WE-1, KA Stations.

The experimental results in Figure 2 show that the detection performance with 5 stations is better than that with 17 stations. The OSE detection accuracy is not only related to the number of stations but also to the distribution of the stations. To explore the relationship between OSE detection accuracy and station distribution, two additional sets of 5-station experiments were conducted in this paper. In each experiment, data from 5 different stations were selected (MO-1, SU-1, BH-1, WE-1, KA combination and ST, MO-2, WA, BH-2, CB combination), and the OSE method was used for detecting the normal modes splitting model. The detection results are shown in Figure 3.

As shown in Figure 3a, the detected single-mode frequencies for *m* = −1 and *m* = 1 are 0.9427 mHz and 0.9458 mHz, with deviations from the PREM theoretical values of 0.433 μHz and 0.328 μHz, respectively. The single-mode signal for *m* = 0 was not detected. In Figure 3b, the detected frequencies for *m* = −1, 0, and 1 are 0.9429 mHz, 0.94467 mHz, and 0.94537 mHz, with deviations from the PREM theoretical values of 0.633 μHz, 0.455 μHz, and −0.102 μHz, respectively. In contrast, Figure 2b detected all single-mode signals with high precision. From Figure 2 and Figure 3, it can be seen that there is a significant correlation between the OSE method’s detection accuracy and station distribution, with a strong dependence on the station combination.

**Figure 4:**
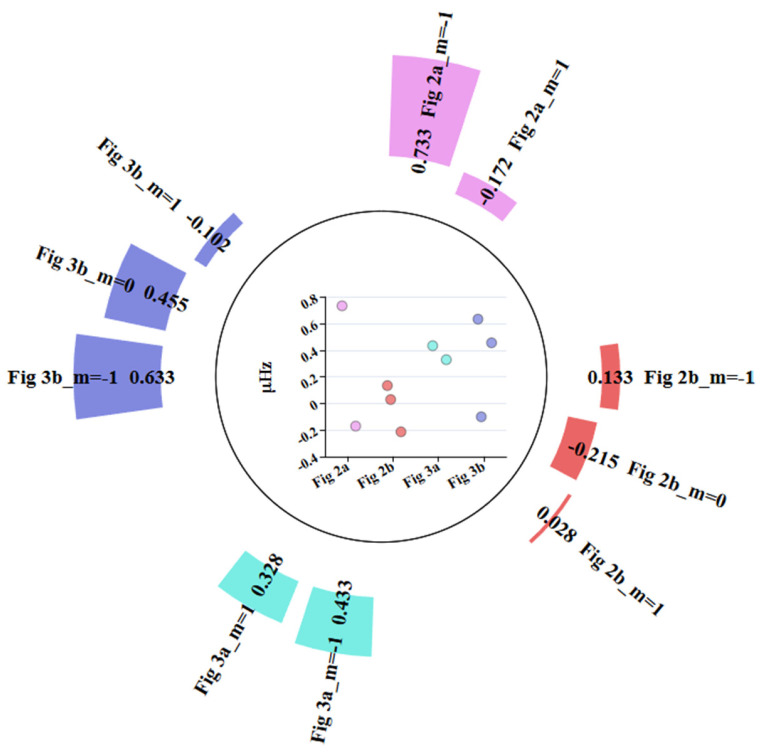
Deviation of the Single-Mode Frequencies Detected in Each Experiment Group from the PREM Theoretical Values.

The detection results of the above experiments are shown in Table 2. The deviations of each set of stacking experiments from the PREM theoretical values are shown in Figure 4. The symbol \ backslash indicates that the signal was not detected.

From the combined statistical results of Table 2 and Figure 4, it can be seen that the detection accuracy of OSE is not directly proportional to the number of stations, which contradicts Equation (8). Furthermore, when the same number of different stations is selected, the OSE detection results also vary significantly. These phenomena suggest that the stations on Earth do not oscillate synchronously, meaning their phases are not the same. Therefore, during stacking, some stations may cancel each other out, leading to large deviations in the OSE detection results when using different numbers of stations or different station combinations with the same number of stations. To improve the accuracy and reliability of the OSE detection results, it is essential to consider the phase information of each station.

### 3.3. NTFT Unbiased Phase Information Extraction

**Figure 5:**
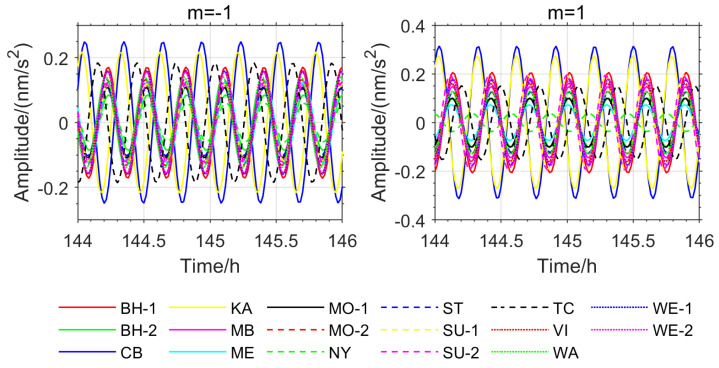
Phase Spectra Extracted by NTFT for the Sumatra Earthquake.

Equations (19) and (20) show that NTFT can unbiasedly determine the instantaneous phase of the normal modes detection model. In this paper, NTFT is used to extract the instantaneous phase information from all stations, as shown in Figure 5. The data used is the superconducting gravimeter data from 50 to 280 h post-earthquake, which has a relatively long duration. However, this paper only presents the instantaneous phase information within the range of 144 to 146 h post-earthquake.

From Figure 5, it can be seen that the instantaneous phases of the 17 stations are not completely identical, with significant differences between some stations. For example, in the *m*= −1 single mode, the CB and KA stations are almost in antiphase with other stations, and the TC station also shows a large phase deviation from the others. In the *m* = 1 single mode, some stations are in antiphase, and some exhibit large phase deviations as well. This further confirms the phenomenon of asynchronous oscillations on Earth. Such a phenomenon causes the inclusion of stations with inconsistent phases to cancel out or weaken the effect of stacking-based resonance methods. Since the phase-inconsistent TC and KA stations were used in Figure 3a,b, the OSE detection results showed larger deviations from the PREM theoretical values. In contrast, the five stations used in Figure 2b have similar instantaneous phases, resulting in detected frequencies for the three multimode oscillations that closely match the theoretical values.

**Figure 6:**
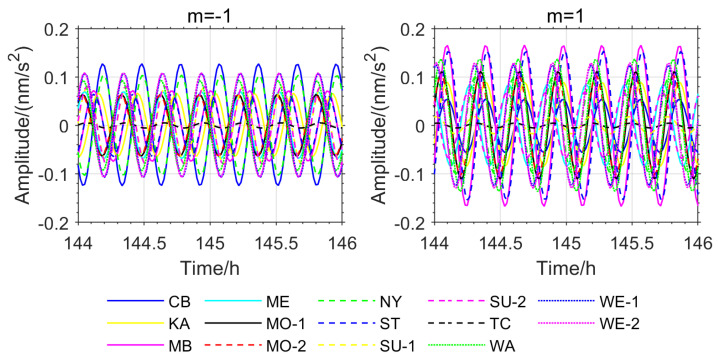
NTFT Extracted Phase Spectrum for the Chile Earthquake.

**Figure 7:**
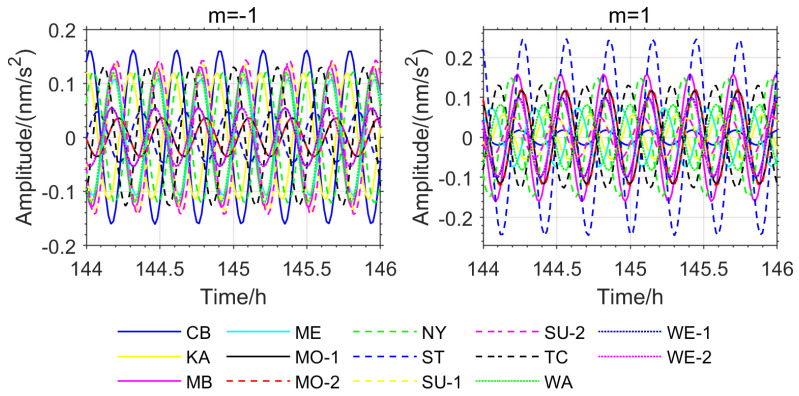
NTFT Extracted Phase Spectrum for the Japan Earthquake.

To eliminate the impact of phase inconsistency caused by station instrument biases and differences in observational conditions, this study further extracted the instantaneous phase information recorded by the used stations during the 2011 Great Tohoku Earthquake (Mw 9.1) in Japan and the 2010 Chilean Earthquake (Mw 8.8). Due to missing data for the VI, BH-1, and BH-2 stations during these two earthquakes on the IGETS website, phase information was ultimately obtained for the remaining 14 stations (Figure 6 and Figure 7).

**Figure 8:**
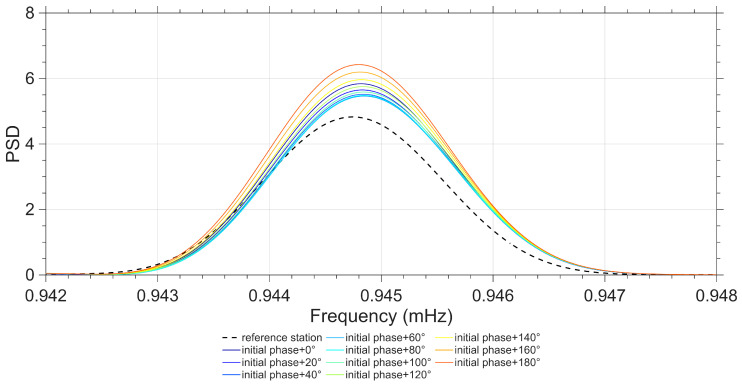
OSE Results of Combinations of Each Simulated Signal with Reference Stations for the _3_S_1_ Mode *m* = 0 Singlet of the Japanese Earthquake Event.

Taking the _3_S_1_ mode *m* = 0 singlet corresponding to the Japanese earthquake event as the research object, we selected a total of 6 stations (MB, ME, SU-1, SU-2, WE-1, and WE-2) as the reference stations and conducted the OSE. The obtained OSE experimental results were processed with NTFT, from which the *m* = 0 singlet signal component was extracted, and the initial phase of this signal was calculated to be 335.09°. Combined with the theoretical frequency of 0.94422 mHz provided by the PREM theoretical model, a series of simulated signals were constructed by setting different initial phase parameters; these simulated signals were individually combined with the reference stations to carry out OSE experiments. The comparative results of the experiments are shown in Figure 8, and the frequency deviation data corresponding to the combinations of simulated signals with different initial phases and reference stations are presented in Table 3.

Based on the experimental comparison results in Figure 8 and the frequency deviation data in Table 3, the phase deviation threshold of the reference stations was scientifically defined: within one signal cycle, if the target frequency accuracy is required to reach 0.01 μHz, the initial phase deviation should be controlled within 20°, and such stations are defined as phase-similar stations; if the target frequency accuracy is required to reach 0.1 μHz, the initial phase deviation can be relaxed to within 40°, and such stations are also defined as phase-similar stations.

The selection of the phase deviation threshold shall be determined according to the accuracy requirements of specific research. This study uniformly selects stations with a phase deviation ≤ 20° as stations with good phase consistency for subsequent analysis.

As shown in Figure 5, Figure 6, Figure 7 and Figure 8, the stations exhibiting phase inversion and large phase deviations differ across different earthquakes, indicating that the phase inconsistency is not caused by data observation. In the Chile earthquake, the CB and NY stations were almost in antiphase with most of the stations, and the MB, ST, and KA stations had large phase deviations from the majority of the stations. In the Japan earthquake, the CB, KA, and NY stations were in antiphase with most of the stations, and the ST and TC stations showed large phase deviations. The ST station, which had large phase deviations during the Chile and Japan earthquakes, was in phase with most of the stations during the Sumatra earthquake. The TC station, which had large phase deviations during the Sumatra and Japan earthquakes, was in phase with most of the stations during the Chile earthquake. This result strongly suggests that the phase differences in the relevant stations are not due to inherent instrument biases or observational environmental factors.

Based on the OSE experiments in this section (Figure 1, Figure 2, Figure 3 and Figure 4, Table 1 and Table 2) and the phase spectrum information (Figure 5, Figure 6 and Figure 7), it can be observed that the OSE detection results vary significantly due to different numbers and distributions of stations. The instantaneous phases of the observation data from different stations are not identical, and these phenomena confirm the presence of phase inconsistency in normal modes. This suggests that the normal modes of different regions of the Earth exhibit temporal differences. Such asynchrony provides a window to observe the physical property differences in various regions of the Earth’s interior. At the same time, these experimental results also indicate that the stacking method must account for phase inconsistency.

## 4. Normal Models Detection Considering Phase Consistency

The OSE method can effectively detect weak signals of normal modes, with the assumption of global synchronous oscillation as its premise. The results in Figure 5, Figure 6 and Figure 7 verify the existence of phase differences between the stations, and this disparity can affect the detection accuracy and sensitivity. To ensure the detection accuracy of weak signals, this paper combines OSE and NTFT, and proposes a high-precision normal modes detection method that accounts for phase consistency. The new method extracts the instantaneous phase spectrum for each station, selects stations with nearly identical instantaneous phases, and then uses the selected stations for OSE detection. By removing stations with phase inconsistencies, this method maximizes the stacking resonance effect, further enhancing the OSE method’s ability to detect weak signals.

**Figure 9:**
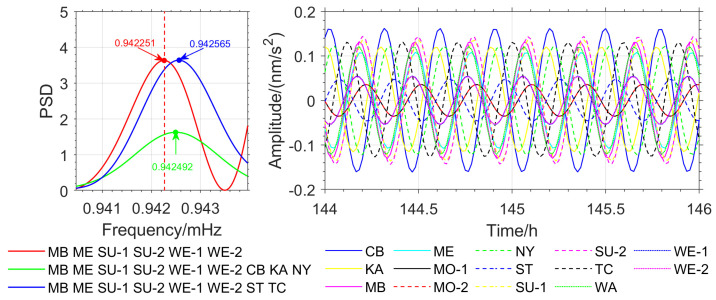
OSE Detection Results for *m* = −1 Single Mode for Three Station Groups in the Japan Earthquake (**Left**) and *m* = −1 Phase Spectrum for Each Station (**Right**).

**Figure 10:**
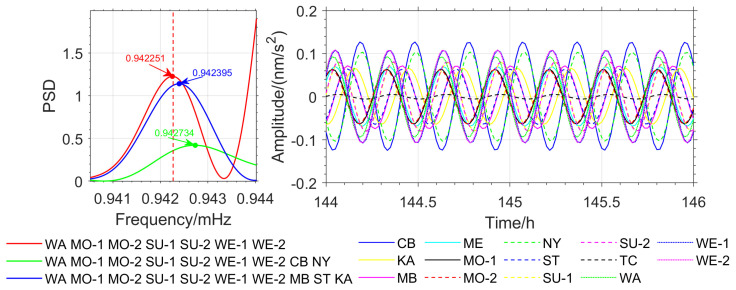
OSE Detection Results for *m* = −1 Single Mode for Three Station Groups in the Chile Earthquake (**Left**) and *m* = −1 Phase Spectrum for Each Station (**Right**).

To further verify the accuracy of the method proposed in this paper, OSE detection based on different seismic events was conducted. Stations with similar phases from the Japan earthquake (MB, ME, SU-1, SU-2, WE-1, and WE-2) were selected, and stations with nearly opposite phases (CB, KA, and NY) as well as stations with significant phase deviations (ST and TC) were added. In the Chile earthquake, stations with similar phases (WA, MO-1, MO-2, SU-1, SU-2, WE-1, and WE-2) were selected, and stations with nearly opposite phases (CB and NY) as well as stations with significant phase deviations (MB, ST, and KA) were added. For the Sumatra earthquake, stations with similar phases (ST, WA, MO-1, MO-2, SU-1, and SU-2) were selected, and stations with nearly opposite phases (CB and KA) as well as the TC station with significant phase deviations were added. The detection of the _3_S_1_ model’s *m* = −1 single-mode is used as an example, and the results are shown in Figure 8, Figure 9 and Figure 10.

**Figure 11:**
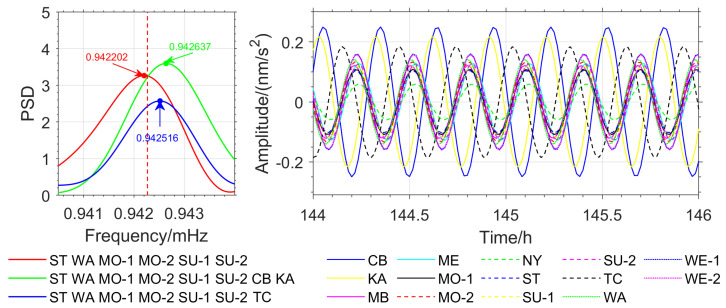
OSE Detection Results for *m* = −1 Single Mode for Three Station Groups in the Sumatra Earthquake (**Left**) and *m* = −1 Phase Spectrum for Each Station (**Right**).

In the Japan earthquake, the OSE detection result for stations with similar phases is 0.942251 mHz, which is in good agreement with the theoretical value. When stations with opposite phases and those with significant phase deviations are introduced, the results are 0.942492 mHz and 0.942565 mHz (see Figure 9), with deviations of 0.241 μHz and 0.314 μHz, respectively. In the Chile earthquake, the detection result for stations with similar phases is 0.942251 mHz. When stations with opposite phases and those with significant phase deviations are introduced, the detection results are 0.942734 mHz and 0.942395 mHz (see Figure 10), with deviations of 0.483 μHz and 0.144 μHz, respectively. In the Sumatra earthquake, the detection result for stations with similar phases is 0.942202 mHz. When stations with opposite phases and those with significant phase deviations are introduced, the detection results are 0.942637 mHz and 0.942561 mHz (see Figure 11), with deviations of 0.435 μHz and 0.359 μHz, respectively. The detection results and deviations are summarized in Table 4.

From Table 4, and Figure 9, Figure 10 and Figure 11, it can be observed that for the three major earthquakes, the OSE detection results for the *m* = −1 single mode of the _3_S_1_ model from stations with nearly identical phases are in good agreement with the PREM theoretical values. Introducing stations with nearly opposite phases or significant phase deviations leads to larger frequency deviations in the detected results, the maximum deviation reaches 0.483 μHz, indicating that phase information affects the high-precision detection of normal modes. In the Japan and Chile earthquakes, introducing stations with nearly opposite phases, as well as introducing stations with significant phase deviations in the Sumatra earthquake, caused a reduction in signal energy in the frequency domain, weakening the resonance amplification effect from stations with nearly identical phases, and thereby diminishing the ability to detect weak signals.

Based on all the experiments conducted, it is concluded that the optimal OSE detection results are obtained from combinations of stations with similar phases, which closely match the PREM theoretical values and allow for the detection of all single modes of the _3_S_1_ model. The inclusion of stations with phase inconsistencies affects the high-precision detection of normal modes, demonstrating that station phase consistency is a key factor when using the OSE method for high-precision normal modes signal detection.

## 5. Conclusions

(1) This study found that 17 stations did not detect the *m* = 0 single mode signal of _3_S_1_, and the detected frequency of the *m* = −1 single mode was 0.943 mHZ, which deviated significantly from the PREM theoretical value of 0.942267 mHZ. Meanwhile, only five stations could detect all three single modes of _3_S_1_, with deviations of 0.133 μHz, 0.215 μHz, and 0.028 μHz between the detected frequencies and the PREM theoretical values, respectively, indicating a high degree of consistency with the theoretical model. By extracting the instantaneous phases of each station using NTFT, it was found that there were phase discrepancies and even phase inversions between the stations. These findings suggest the existence of phase inconsistency (or asynchronous oscillation) in normal modes of the Earth.

(2) Experiments (Figure 2, Figure 3, Figure 9, Figure 10 and Figure 11) show that the inclusion of stations with asynchronous oscillations with phase differences greater than 20°, on one hand, reduces the frequency detection accuracy of OSE. Specifically, for the Japan earthquake (11 March 2011), Chile earthquake (27 February 2010), and Sumatra earthquake (26 December 2004), nearly anti-phase stations and stations with significant phase deviation have deviations of 0.241 μHz, 0.483 μHz, 0.435 μHz and 0.314 μHz, 0.144 μHz, 0.359 μHz, respectively, relative to phase-consistent stations. On the other hand, it weakens OSE’s ability to detect weak signals. Therefore, to improve the detection accuracy and sensitivity of the stacking method, phase inconsistency must be considered.

(3) This paper proposes a method that high-precision detection of normal modes Signals with Consideration of Phase Consistency. This method combines the unbiased phase revelation capability of NTFT and the weak signal detection ability of OSE. Experimental results indicate that the new method has certain advantages in high-precision detection of weak signals and provides high-precision baseline data for further constraining and refining Earth models.

The asynchronous oscillation phenomenon presented in this paper offers a window to observe the physical property differences in various regions of Earth’s interior. Future work will use globally distributed broadband seismometer data to reveal the spatial distribution characteristics of asynchronous oscillations, providing new ideas and methods for uncovering the small-scale structure of Earth’s interior, especially in the deep Earth.

## Figures and Tables

**Table 1:** Station Information.

Station Name	Number	Instrument Name	Longitude (°)	Latitude (°)	Elevation (m)
Wettzell	WE-1 WE-2	GWR CD029_U GWR CD029_L	12.878	49.144	613.700
Walferdange	WA	OSG-CT40	6.1528	49.6647	295.000
Vienna	VI	GWR C025	16.3579	48.2493	192.440
TIGO Concepcion	TC	GWR RT038	−73.025649	−36.84376	156.140
Sutherland	SU-1 SU-2	D037_U D037_L	20.8109	−32.3814	1791.000
Ny-Alesund	NY	GWR C039	11.86717	78.93061	43.000
Moxa	MO-1 MO-2	CD034_U CD034_L	11.6160	50.645	455.000
Metsahovi	ME	GWR T020	24.3958	60.2172	55.600
Strasbourg	ST	GWR C026	7.6838	48.6217	180.000
Membach	MB	GWR C021	6.0066	50.6093	250.000
Kamioka	KA	GWR T016	137.3084	36.4253	358.000
Canberra	CB	GWR C031	149.00766	−35.32064	762.749
Bad Homburg	BH-1 BH-2	GWR CD030_U GWR CD030_L	8.6113	50.2285	190.000

**Table 2:** OSE Detection Results for Each Experiment Group.

Splitting Modes	m = −1 (mHZ)	m = 0 (mHZ)	m = 1 (mHZ)
PREM	0.942267	0.944215	0.945472
Stations used in Figure 2a	0.943	\	0.9453
Stations used in Figure 2b	0.9424	0.944	0.9455
Stations used in Figure 3a	0.9427	\	0.9455
Stations used in Figure 3b	0.9429	0.94467	0.94537

**Table 3:** Phase Differences in the _3_S_1_ Model (*m* = −1) Between Stations for the Three Major Seismic Events.

Phase Difference Relative to the Reference Signal (°)	Frequency Deviation of OSE Results for Combinations with Reference Stations (μHz)
0	0.0625
20	0.0966
40	0.1067
60	0.1087
80	0.1208
100	0.1167
120	0.1207
140	0.0966
160	0.0725
180	0.0621

**Table 4:** Impact of Stations with Phase Deviations on Detection Accuracy.

Seismic Events	Results for Stations with Nearly Identical Phases (mHz)	Results for Stations with Nearly Opposite Phases (mHz)	Deviations for Stations with Nearly Opposite Phases (μHz)	Results for Stations with Significant Phase Deviations (mHz)	Deviations for Stations with Significant Phase Deviations (μHz)
Japan Earthquake (11 March 2011)	0.942251	0.942492	0.241	0.942565	0.314
Chile Earthquake (27 February 2010)	0.942251	0.942734	0.483	0.942395	0.144
Sumatra Earthquake (26 December 2004)	0.942202	0.942637	0.435	0.942561	0.359

## Data Availability

SG data products were downloaded freely from the IGETS data base at GFZ Potsdam (http://igets.gfz-potsdam.de/, accessed on 2 November 2024).

## Abbreviations

The following abbreviations are used in this manuscript:

NTFT	Normal Time-Frequency Transform
OSE	Optimal Sequence Estimation
